# Predictive modeling and x-ray crystallography for mechanistic understanding of GO DNA repair

**DOI:** 10.1063/4.0001027

**Published:** 2025-10-27

**Authors:** Martin P Horvath, Melody Malek, Danielle Yama, Carlos Trasviña-Arenas, Sheila S David

**Affiliations:** 1 University of Utah, Salt Lake City, Utah, USA; 2 University of California Davis, Davis, California, USA; 3 Cenvistav, Mexico City, Ciudad de México, Mexico

## Abstract

Background: GO DNA repair protects against mutations that otherwise result from oxidative damage to guanine. The 8-oxo-7,8-dihydro-guanine lesion (OG) pairs with C and A, a source of ambiguity that explains GC → TA mutations when GO DNA repair is compromised. The OG:C lesion is recognized and processed by OGG1 to remove the OG base and thus initiate base excision repair (BER). The OG:A lesion is recognized and processed by MutY/MUTYH to remove the undamaged but information-defective A base.

Case study 1: Predictive modeling (prior AlphaFold) and biochemical experiments showed that OGG1 and MutY DNA glycosylases have very different active sites with OGG1 accommodating large, bulky transition state mimics and leaving groups while MutY/MUTYH is by comparison restricted to smaller transition state mimics (Yuen, 2019). We have now determined x-ray crystal structures for OGG1 in complex with transition state mimics (Image 1), thereby enabling direct comparison for these predicted models.

Case study 2: Predictive modeling with Colabfold provided insight for the evolution of MutY-like enzymes in archaea (MutYA)s. Functional characterization has shown representative MutYAs can substitute for MutY when expressed in E. coli. Colabfold predicted structures of MutYAs were recognizably similar with the structures of MutY from bacteria and mammals (Image 2), but this similarity was restricted to the N- terminal catalytic domain. The C-terminal domains are distinctly different comparing MutYA and MutY/MUTYH. The C-terminal domain of MutY/MUTYH comprises a Nudix protein fold and resembles the OG-phosphatase MutT/MTH1 that hydrolyzes OG-triphosphates. By contrast, the C-terminal domain from MutYA comprises a winged helix-turn-helix fold found in many transcription factors and other DNA-binding proteins. This predicted winged helix-turn-helix fold found in our MutYA models was also revealed recently in the x-ray crystal structure of MutYX, a MutY from the bacteria *Eggerthella* sp. strain YY7918 (Trasviña-Arenas, in press). The combination of prediction and experimental structures provides exciting insight for the evolution of MutY.

